# Chemical Screens Identify Drugs that Enhance or Mitigate Cellular Responses to Antibody-Toxin Fusion Proteins

**DOI:** 10.1371/journal.pone.0161415

**Published:** 2016-08-24

**Authors:** Antonella Antignani, Lesley Mathews Griner, Rajarshi Guha, Nathan Simon, Matteo Pasetto, Jonathan Keller, Manjie Huang, Evan Angelus, Ira Pastan, Marc Ferrer, David J. FitzGerald, Craig J. Thomas

**Affiliations:** 1 Laboratory of Molecular Biology, Center for Cancer Research, National Cancer Institute, National Institutes of Health, Bethesda, Maryland 20892–4264, United States of America; 2 Division of Preclinical Innovation, National Center for Advancing Translational Sciences, National Institutes of Health, Rockville, Maryland, 20850, United States of America; University of PECS Medical School, HUNGARY

## Abstract

The intersection of small molecular weight drugs and antibody-based therapeutics is rarely studied in large scale. Both types of agents are currently part of the cancer armamentarium. However, very little is known about how to combine them in optimal ways. Immunotoxins are antibody-toxin gene fusion proteins engineered to target cancer cells via antibody binding to surface antigens. For fusion proteins derived from Pseudomonas exotoxin (PE), potency relies on the enzymatic domain of the toxin which catalyzes the ADP-ribosylation of EF2 causing inhibition of protein synthesis leading to cell death. Candidate immunotoxins have demonstrated clear value in clinical trials but generally have not been curative as single agents. Therefore we undertook three screens to discover effective combinations that could act synergistically. From the MIPE-3 library of compounds we identified various enhancers of immunotoxin action and at least one major class of inhibitor. Follow-up experiments confirmed the screening data and suggested that immunotoxins when administered with everolimus or nilotinib exhibit favorable combinatory activity and would be candidates for preclinical development. Mechanistic studies revealed that everolimus-immunotoxin combinations acted synergistically on elements of the protein synthetic machinery, including S61 kinase and 4E-BP1 of the mTORC1 pathway. Conversely, PARP inhibitors antagonized immunotoxins and also blocked the toxicity due to native ADP-ribosylating toxins. Thus, our goal of investigating a chemical library was justified based on the identification of several approved compounds that could be developed preclinically as ‘enhancers’ and at least one class of mitigator to be avoided.

## Introduction

Antibody-based therapeutics show great promise for the treatment of patients with cancer [1]. Ideally, the chosen antibody binds to surface antigens on malignant cells and not to healthy tissues. One successful strategy for producing therapeutic antibodies is the construction of antibody-toxin fusion proteins, also known as recombinant immunotoxins [2, 3]. Immunotoxins produced from truncated versions of Pseudomonas exotoxin (PE) kill cells via the ADP-ribosylation of elongation factor 2 leading to inhibition of protein synthesis [2, 3]. Several immunotoxins have been evaluated already in clinical trials with some striking results including a high percentage of complete remissions in patients diagnosed with hairy cell leukemia when treated with immunotoxins targeting surface-expressed CD22 [4–6]. However, the same immunotoxins produced fewer responses in other CD22-positive B-cell malignancies such as CLL or NHL [4]. Similarly, an immunotoxin targeting mesothelin (SS1P) produced few objective responses when evaluated as a single agent in patients diagnosed with mesothelioma [7, 8]. To achieve maximum benefit, it is likely that immunotoxins will need to be administered in combination with small molecular weight drugs or other types of therapies. Ideally, suitable combinations can be identified that are synergistic for killing cancer cells while avoiding increased systemic toxicity. To identify effective immunotoxin-drug combinations we devised screens using both epithelial (KB3-1) and hematological (Nalm-6) cell lines. KB3-1 cells were incubated with the SS1P immunotoxin while Nalm-6 cells were treated with HA22, the immunotoxin targeting CD22. The goal was to find compounds that enhanced immunotoxin activity, with a preference for those drugs that were approved already for human use. Further, the screen should also identify potential mitigators, i.e. combinations to be avoided. Here we report on the outcomes of screens using the MIPE-3 small molecule library of 'cancer focused' compounds containing both approved and investigational drugs [9]. Immunotoxins at fixed concentrations were added to cells that were treated with eleven concentrations of each drug spanning a 4.5 log_10_ range. To avoid trivial differences related to immunotoxin design, both immunotoxins, SS1P and HA22, were constructed as disulfide-stabilized antibody Fvs joined with truncated PE38 from Pseudomonas exotoxin [3]. Purified immunotoxins of clinical grade were used in both screens. As outlined below, the screening effort was successful in identifying a number of approved compounds that enhanced the action of both immunotoxins. Likewise, mitigators were also identified, including, prominently, PARP inhibitors. And more generally our results confirmed the utility of screening drugs and antibody-based therapeutics using cell-based viability assays in a multiwell format.

## Results

### Chemical screens identified both enhancing and mitigating drugs

**Fig 1:**
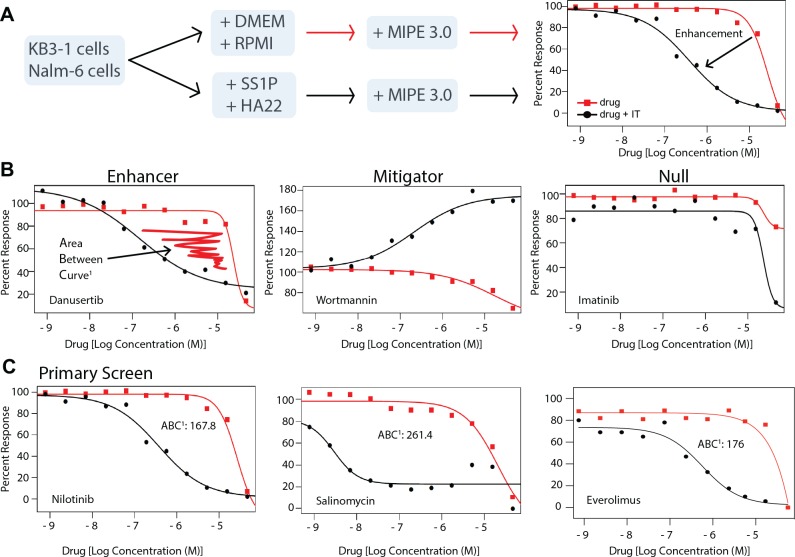
A chemical genetics screen that identifies enhancers and mitigators of immunotoxin-based cellular toxicity. **A.** A schematic outlines the screening flow. Either KB3-1 cells or Nalm-6 cells were treated with media or the appropriate immunotoxin (SS1P for KB3-1 or HA22 for Nalm-6) at a predetermined concentration that was sublethal to the plurality of cells. A quantitative 'high throughput screen' of the MIPE-3.0 library of approved and investigational drugs was conducted generating an 11-point dose response curve for all agents. The curves for each agent alone (red line) or plus immunotoxin (black line) were then compared in order to identify enhancement or mitigation. **B.** An illustration of a strong enhancer (danusertib), mitigator (wortmannin) and an agent with null effect (imatinib). These curves were recorded in the KB3-1 screen. **C.** The primary screening outcomes for nilotinib, salinomycin and everolimus. The curves for nilotinib and salinomycin were recorded in the KB3-1 screen while the everolimus curve is taken from the Nalm-6 screen. The Area Between the Curve^1^ score is derived from the differences between the area under the curve (AUC) for each dose response as computed using the trapezoidal rule (see the SI for methods). Primary screening assays were read at 48 hours.

To identify advantageous drug combinations to co-administer with an antibody-based immunotoxin, we used curve shift analysis rather than a full matrix screening approach. This was necessitated because solvents used for acoustic dispensing of drugs, inactivated the immunotoxin protein, which was discovered while conducting preliminary experiments. Accordingly, dose response curves were generated for each compound of the MIPE-3 chemical library in the absence and presence of a fixed sublethal concentration of immunotoxin. The analysis of the screen (the calculated Area Between the Curves, ABC) determined if the immunotoxin enhanced, mitigated or had no effect on the cytotoxicity of candidate drugs (Fig 1A). The MIPE 3.0 library was described recently and consists of 459 agents including oncology-focused and mechanistically important drugs of clinical relevance [9]. Further, in an effort to distinguish general enhancers from cell line- or immunotoxin-specific effects, screens were conducted with two immunotoxins and two cell lines. Specifically, KB3-1 cells were treated either with media or the immunotoxin, SS1P (Table 1), targeting surface mesothelin, and then dispersed into wells containing the constituents of the MIPE-3 library dispensed over a 4.5 log_10_ concentration range. Or Nalm-6 cells were treated with the HA22 immunotoxin (Table 1), targeting CD22, and likewise dispersed into drug containing wells. (Table 1 lists the immunotoxins and targets used in the various screening and confirmation assays). One dataset was generated with SS1P and two datasets were generated with HA22 (a high set at 200 ng/ml and a low one at 20 ng/ml). The outcomes of these screens are included in a supporting information file (S1 Table) and are publically available via the PubChem database (https://pubchem.ncbi.nlm.nih.gov/)(summary AID 1159514). While our major focus was the identification of enhancing compounds, it was also important to discover mitigators: i.e. compounds to be avoided as immunotoxin 'partners'. Enhancers and mitigators (Fig 1B) were identified for both immunotoxins through visual examination of the complete response curves for each drug and by calculating an ABC score as the difference between the drug alone and the drug plus immunotoxin curves (see the supporting information for a description of the ABC scoring method). Examples of enhancers (danusertib), mitigators (wortmannin) and agents with null effect (imatinib) are shown in Fig 1B. By using two immunotoxins and two target cell lines, it was possible to identify both 'general' enhancers and 'cell-specific' enhancers (Fig 1C and S1 Fig). General enhancers enhanced both immunotoxins on both cell lines (Fig 1C); while cell-specific enhancers increased either HA22 activity for Nalm-6 cells (S1A Fig) or SS1P activity for KB3-1 cells (S1B Fig) but did not enhance in both cell lines. Cell-specific enhancers may be of interest in dissecting pathway differences utilized by specific immunotoxins. Examples of agents with preferential activity for either HA22 or SS1P included: U-73122 or Abiraterone that were enhanced by HA22 (S1A Fig) and Tozasertib or SNS-314 that were enhanced by SS1P on KB3-1 cells (S1B Fig).

**Table 1:** 

Immunotoxin name	Surface target	Cancer target
HA22	CD22	B-cell malignancies
SS1P	Mesothelin	Mesothelioma, cancers of the pancreas, lung and ovary
HB21PE40	Transferrin receptor	Not applicable

As well as enhancers, mitigators were also identified (Table 2). These included selected phosphoinositide 3-kinase and AKT inhibitors (S2A Fig). However, a prominent "family" of mitigators was identified within the group of compounds termed PARP inhibitors: olaparib, veliparib and rucaparib each reduced immunotoxin action substantially (Table 2 and S2B Fig).

**Table 2:** 

Agent	Pharmacology	KB3-1 ABC Score	Nalm-6 ABC Score
**Enhancers**			
Everolimus	mTOR1 inhibitor, FKBP-12	-68^#^	-176
Salinomycin	Wnt Signaling inhibitor	-261	-83
Nilotinib	Bcr-Abl, DDR1, cKit, PDGFR inhibitor	-168	-152
Verapamil	Ca channel, CYP3A4, Dopamine D2 antagonist	-118	-180
Niguldipine	L-type Ca channel blocker	-137	NC
Mifepristone	Progesterone, Glucocorticoid R antagonist	-108	-37
Danusertib	Aurora K A/B/C inhibitor	-101	-100
GB83	PAR2 antagonist	-89	-85
GSK-1904529A	IGF1-R inhibitor	-184	-132
Serdemetan	MDM2 inhibitor	-82	61
**Mitigators**			
Wortmannin	PI3K inhibitor	211	NC
GSK-690693	AKT-inhibitor	414	NC
OSI-027*	mTORC1/mTORC2 inhibitor	171	154
Olaparib**	PARP1/2 inhibitor	337	1362
Torin1***	mTOR inhibitor	175	-59
AZD-8055***	mTOR inhibitor	205.5	-48
KU-0063794***	mTOR inhibitor	136	-31

#This score is derived from the enhancing portion of the curve shown in S4A Fig.

*OSI-027 and other mTOR kinase inhibitors were scored as weak mitigators relative to the strong mitigation seen with PARP inhibitors such as

**olaparib and rucaparib. ** For the nicotinamide mimetic PARP inhibitors, scores of greater than 1000 were noted routinely.

*** In Nalm-6 cell line, the curves with or without the immunotoxin were indistinguishable.

### Confirmation of screening data

**Fig 2:**
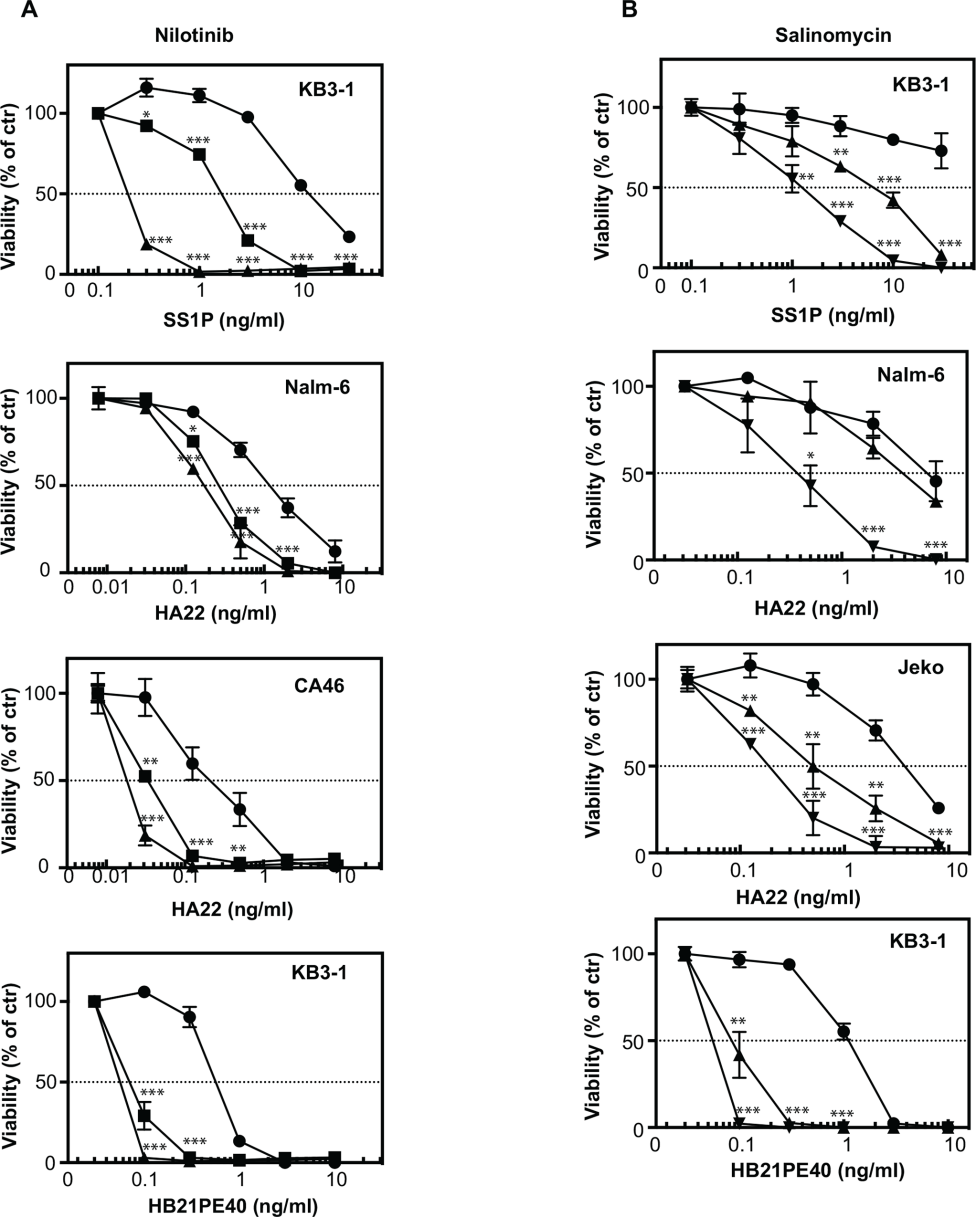
Confirmation of enhancer activity for nilotinib and salinomycin combinations. Cells were treated for 72 hours in a dose dependent manner with immunotoxins in the absence (closed circle) or in the presence of different concentrations of nilotinib (**A**) or they were treated for 48 hours in a dose dependent manner with immunotoxins in the absence (closed circle) or in the presence of different concentrations of salinomycin (**B**). Nilotinib was tested at 1 μM (closed square) and 2 μM (closed triangle) Salinomycin was tested at 0.05 μM (closed tringle) and 0.1 μM (inverted closed triangle) in Nalm-6 and Jeko cells with HA22 and at 0.025 μM (closed triangle) and 0.05 μM (inverted closed triangle) in KB3-1 cells with SS1P and HB21PE40. Data are from at least two independent experiments in triplicate. Error bars display SD value. Two tailed unpaired t-tests were performed at each concentration point. Two tailed unpaired t-tests were performed comparing the viability at each concentration of immunotoxin alone versus the same concentrations plus the drug with p<0.05 significant. *p<0.05, **p<0.01, ***p<0.001.

Screens identified key compounds because of shifts in drug responses caused by the presence of immunotoxin. Left shifts (reported here with negative numbers for ABC values) were due to enhancement effects. To confirm these, additional experiments were conducted using multiple concentrations of each immunotoxin. Drugs with a strong enhancement effect for both the KB3-1 and Nalm-6 cell lines were of particular interest. Several agents possessed this pan-activity including nilotinib, salinomycin and everolimus (Fig 1C). Nilotinib (Tasigna®, AMN107) is a tyrosine kinase inhibitor approved for the treatment of chronic and accelerated phase Philadelphia chromosome-positive chronic myelogenous leukemia in patients resistant or intolerant to prior therapy with imatinib [10]. Nilotinib binds to and inhibits the kinase domain of ABL/BCR-ABL but also interacts with other cancer-expressed kinases including DDR1, KIT and PDGFR [11, 12]. Nilotinib was among the agents most significantly enhanced by the presence of the immunotoxins in the chemical screen with an ABC score of -167.8 in the KB3-1 screen and -152.5 in the Nalm-6 screen (Table 2). In confirmatory studies Nilotinib enhanced the cytotoxic activity of SSIP for KB3-1 cells by more than 10-fold, the activity of HA22 toward Nalm-6 and CA46 cells by ~5-fold and that of HB21PE40 (an immunotoxin targeting the transferrin receptor–see also Table 1) in KB3-1 cells by ~ 10-fold (Fig 2A). Surprisingly, other BCR-ABL-targeting drugs did not enhance the activity of either immunotoxin including imatinib (Gleevec)(Fig 1B). We reasoned, therefore, that the strong action of nilotinib relative to imatinib was the result of its targeting distinct kinase(s) unrelated to ABL or BCR-ABL.

The strongest enhancement within the KB3-1 screen was recorded with salinomycin within the KB3-1 screen (ABC score of -261.4). Salinomycin also scored as an enhancer in the Nalm-6 screen (ABC score -83.2)(Table 2). This agent, therefore, was a compound of interest despite the fact that it is not currently approved for human use. Salinomycin has been reported to have cytotoxic activity against cancer stem cells due to antagonism of the Wnt/Beta-catenin pathway and also as an anti-microbial agent [13–15]. In confirmation studies, salinomycin was found to enhance strongly (by 10-fold or more) the activity of SSIP, HA22 and HB21PE40 in several cell lines (Fig 2B). These data confirmed and expanded the screening results. For nilotinib and salinomycin, substantial enhancement was noted with at least three immunotoxins targeting different surface antigens and with several cell lines. Neither compound alone had a major adverse effect on cell viability. (S3A and S3B Fig). Thus we conclude that these compounds are 'general' enhancers of immunotoxin activity that act synergistically to increase cell killing.

### Immunotoxins in combination with everolimus block key signaling elements and synergistically delay tumor growth *in vivo*

**Fig 3:**
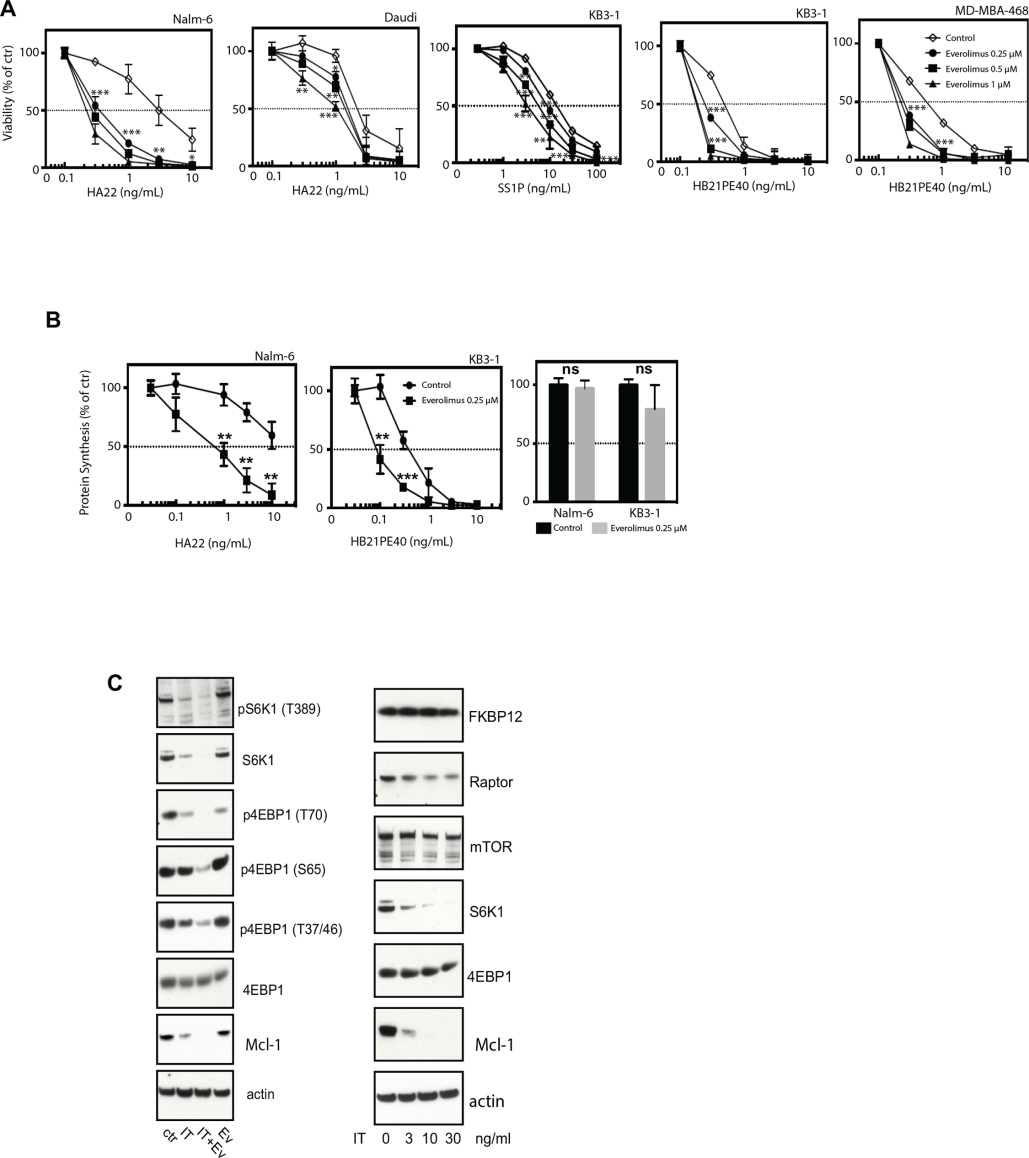
Everolimus enhances immunotoxin action through coordinated down-regulation of protein synthesis and is also effective in a tumor xenograft model. **A.** Confirmation results demonstrating that everolimus enhances the cellular toxicity of the HA22, SS1P and HB21PE40 immunotoxins in multiple cell types. Data were compared using two tailed unpaired t-tests where the viability at each concentration of immunotoxin alone versus the same concentrations plus the drug was compared. p<0.05 significant. *p<0.05, **p<0.01, ***p<0.001. **B.** The combination enhances the down regulation of protein synthesis as measured by incorporation of 3H-leucine into cells. Data were compared using two tailed unpaired t-tests where the viability at each concentration of immunotoxin alone versus the same concentrations plus the drug was compared. p<0.05 significant. *p<0.05, **p<0.01, ***p<0.001. **C.** Left panel: analysis of key elements of the mTORC1 signaling pathway including Western blot analysis of S6K1 and pS6K1; p4EBP1 (several sites), 4EBP1 and Mcl-1 following treatment with everolimus (0.5 μM), the HB21PE40 immunotoxin (10ng/ml), the combination or a DMSO control. The blots on the right represent the detection of FKBP12, Raptor, mTOR, S6K1, 4EBP1 and Mcl-1 following increasing concentrations of the HB21PE40 immunotoxin.

The primary screen demonstrated strong enhancement by the mTOR inhibitor, everolimus, in both the KB3-1 and Nalm-6 screens (ABC scores of -68.3 and -176.7 in the KB3-1 and Nalm-6 screen, respectively (Table 2). Note the ABC score in KB3-1 is artificially high due to the curve’s starting max response value (S4A Fig). Everolimus is approved for a number of oncologic indications [16, 17] and, because of this, it was among the primary hits chosen for further study. Moreover, everolimus selectively targets the mTORC1 pathway, which controls protein synthesis through the phosphorylation of the eIF4E-binding protein 1 (4E-BP1) and S6 kinase 1 (S6K1) [18]. Immunotoxin-mediated inactivation of EF2 together with alterations in the protein synthetic machinery by everolimus, provided an intriguing focus for the observed synergy. Therefore we undertook a more detailed investigation of these two agents in combination. First we evaluated a variety of everolimus-immunotoxin combinations and noted strong enhancement (range of 3-10-fold) in all cell lines, confirming everolimus as a 'general' enhancer of immunotoxin action (Fig 3A). Further at concentrations up to 1 μM, everolimus, when added as a single agent, reduced cell viability by no more than 25% (S3C Fig). Thus we detected enhancement even when everolimus by itself was only minimally cytotoxic. Also, we established that the combination of everolimus (0.25μM) and immunotoxins reduced protein synthesis to lower levels than either agent alone (Fig 3B). In fact, everolimus alone caused only a slight decrease in protein synthesis (Fig 3B). Of interest, rapamycin, another mTORC1 inhibitor, had a qualitatively similar enhancing activity to everolimus (data not shown).

A consideration of key mechanistic elements of mTORC1 signaling was insightful. The eukaryotic initiation factor 4E-binding proteins (4EBPs) and S6 kinase (S6K) are the main regulators of protein synthesis downstream of mTORC1. mTORC1 directly phosphorylates the binding protein, 4EBP1, releasing its inhibitory effect on the translation initiation factor eIF4E, promoting cap-dependent mRNA translation [19]. S6K indirectly affects protein synthesis by phosphorylating the ribosomal protein S6, which regulates transcription of ribosomal proteins [19]. Everolimus reduces the phosphorylation of both 4EBP1 and S6K increasing the interaction between eIF4E and 4EBP1 and reducing ribosome biogenesis with the consequent reduction in protein synthesis [19,20]. We examined the protein levels and phosphorylation of 4EBP1 and S6K in cells treated with the immunotoxins, everolimus or a combination of both. To highlight the cooperation between the two reagents, we chose a short treatment window (24h) and used concentrations of the immunotoxin and everolimus that caused minimal single-agent loss of cell viability. Examination of the S6K1 protein revealed that the immunotoxin by itself dramatically reduced the intracellular level of this kinase (which, to our knowledge, has not been reported before), accounting for the decreased phosphorylated form of this enzyme (Fig 3C left panel). Strikingly, in the combination, the addition of everolimus eliminated all detectable S6K1. Further, immunotoxin-mediated downregulation was dose dependent and specific for S6K1. (Fig 3C right panel). We conclude that immunotoxin-mediated loss of S6K was critical for the toxin contribution toward inhibition of the mTORC1 pathway. As a cellular marker for short-lived proteins that are rapidly degraded during protein synthesis inhibition, levels of the prosurvival protein Mcl-1 were monitored as reported previously [21, 22]. Of interest we found that Mcl1 was reduced with the immunotoxin alone but there was complete loss following co-administration of immunotoxin and everolimus (Fig 3C).

A second major element of mTOR signaling involves the phosphorylation of 4EBP1 by mTORC1 [20]. On KB3-1 cells, except for Thr70, neither the immunotoxin nor everolimus alone caused detectable reductions in the phosphorylation of 4EBP1. However, 4EBP1 phosphorylation was greatly reduced in the cells treated with the combination, even on reported rapamycin-resistant sites, Thr37/46 [23](Fig 3C left panel). Taken together, the combined actions of the immunotoxins and everolimus synergistically blocked phosphorylation of key residues on 4EBP1. Thus, everolimus enhances toxin-mediated inhibition of protein synthesis (Fig 3B) and the immunotoxin enhances everolimus-mediated inhibition of downstream targets of mTORC1 (Fig 3C left panel), supporting a synergistic action between these two compounds.

**Fig 4:**
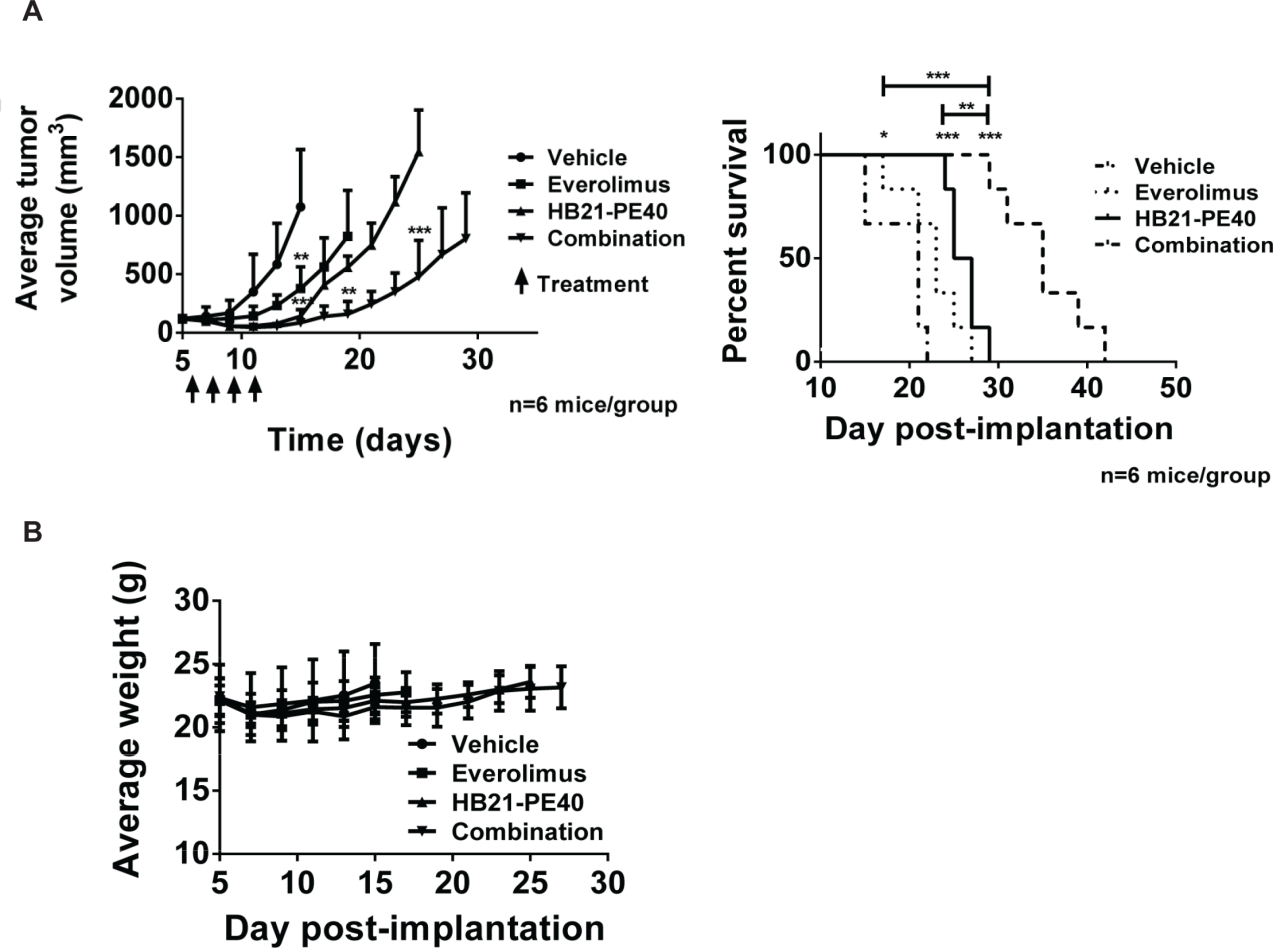
The combination everolimus and immunotoxin is effective in a tumor xenograft model. **A.**Tumor volumes were compared by unpaired two-tailed t-test at each measurement. For the Kaplan-Meyer plot, significance was assessed by log-rank test. **B.** Weight measurements of mice for the experiment in Fig 4A.

While tissue culture experiments confirmed screening results and provided mechanistic insight into everolimus-mediated enhancement of immunotoxins, it was also important to test combination treatments in vivo. To characterize the activity of everolimus in tumor models, a xenograft model was devised whereby KB3-1 tumors were injected subcutaneously and then subjected to treatments that included, everolimus alone, immunotoxin (HB21-PE40) alone or a combination of both. A preliminary experiment indicated that everolimus at 10 mg/kg and immunotoxin at 150 μg/kg were tolerated well when injected as single agents (S4B Fig). The combination however, led to weight loss and death in 40% of mice. However, when tumor growth was monitored in the surviving mice, combination treatments appeared to be superior to either treatment alone (S4B Fig). Therefore, a second experiment was devised with everolimus administered at 5 mg/kg and immunotoxin at 100 μg/kg. All mice in all groups (n = 6 per group) survived with no evidence of weight loss (Fig 4B). Results indicated that there was a significant antitumor response with immunotoxin or everolimus as single agents. However, a superior result was achieved with the combination (Fig 4A). The drug combination not only inhibited tumor growth but produced a superior extension of survival relative to either drug administered alone.

### PARP inhibitors mitigate the actions of immunotoxins and toxins

**Fig 5:**
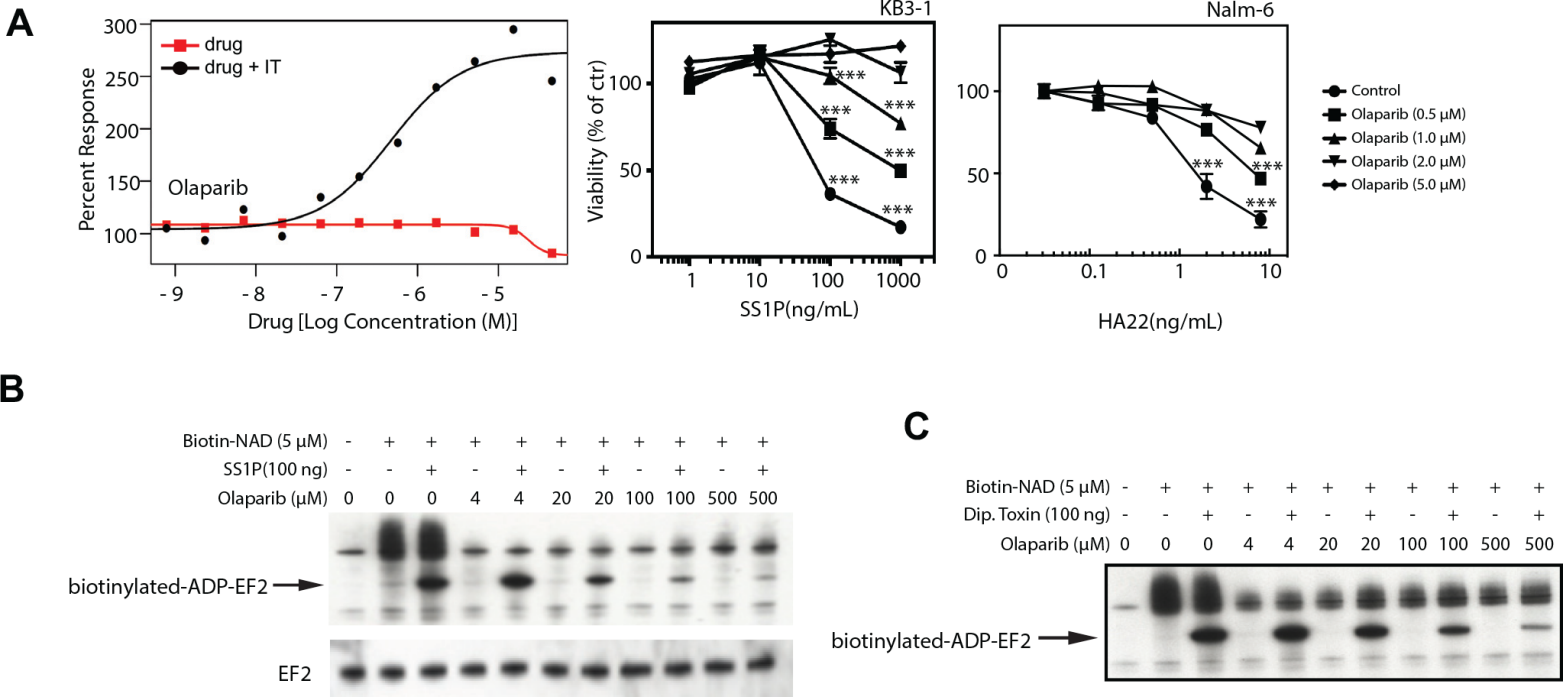
Olaparib mitigates the actions of PE-based immunotoxins and diphtheria toxin. **A.** The primary screening and confirmation results demonstrating that olaparib mitigates the cellular toxicity of the SS1P immunotoxins in KB3-1 cells. The curves for olaparib alone (red line) or plus SS1P (black line) in the primary screening were compared. Confirmatory data were then compared using two tailed unpaired t-tests where the viability at each concentration of immunotoxin alone versus the same concentrations plus the drug was compared. p<0.05 significant. *p<0.05, **p<0.01, ***p<0.001. **B.** Western Blot analysis of biotinylated EF2 following treatment of cell lysates with biotin-NAD, SS1P and increasing concentrations of olaparib. Upper panel shows detection of biotin levels via streptavidin-HRP while the lower panel is probed with an anti-EF2 antibody. C. Western Blot analysis of biotinylated EF2 following treatment of cell lysates with biotin-NAD, diphtheria toxin and increasing concentrations of olaparib. Bands show detection of biotin levels via a streptavidin-HRP.

When analyzing screen results, right curve shifts indicated antagonism: and this was noted with immunotoxins and PARP inhibitors. In fact, multiple PARP inhibitors acting in a dose-dependent manner and from distinct chemical classes were identified as potential mitigators of immunotoxin action. This suggested a common mechanism of action. Olaparib is approved for BRCA-mutated ovarian cancer patients and Phase III testing is in progress for breast cancer patients with BRCA1/2 mutations. PARP inhibitors are also being evaluated clinically in combination with other agents. Because, PE-based immunotoxins exert their toxic action via the ADP-ribosylation of EF2, it was conceivable that the target of each PARP inhibitor was the immunotoxin itself. Further, Merrill and colleagues have suggested that PARP-like inhibitors can act as protective agents against PE and cholix toxins [24]. To explore the possibility that PARP inhibitors were blocking immunotoxin-mediated ADP-ribosylation directly, we undertook two kinds of studies. In cell-based assays, olaparib protected KB3-1 cells from SS1P-mediated toxicity (Fig 5A and S2B Fig) and Nalm6 cells from HA22 (Fig 5A). Then in cell-free studies we assessed the ability of olaparib to inhibit immunotoxin-mediated labeling of EF2 with a biotinylated NAD probe. This system was described previously as a cell-free assay for monitoring the ADP-ribosylating activity of PE-derived immunotoxins [25]. By monitoring the amount of NAD-labeled EF2 in the presence of increasing doses of olaparib we confirmed that PARP inhibitors blocked the ADP-ribosylating function of the SS1P immunotoxin (Fig 5B).

As immunotoxins utilize the catalytic functions and domains of native toxins including diphtheria toxin and pseudomonas exotoxin A [26] it was of interest to examine whether PARP inhibitors blocked a similar catalyzed reaction by the toxins themselves. To test this, we incubated native toxins with increasing concentrations of olaparib: and found that the drug did indeed inhibit the ADP-ribosylating function of both diphtheria toxin (Fig 5C) and PE (data not shown). Finally, PARP inhibitors scored as stronger mitigators in the KB3-1 screen than in the 'low does HA22' Nalm-6 screen. To characterize the activity of olaparib in Nalm6 cells in greater detail, we incubated cells with increasing concentrations of drug in the presence of HA22 or DT. From both assays, protection of cells was clearly evident at concentrations of 2 μM and above of olaparib (Fig 5A and data not shown). From these data we conclude that PARP inhibitors block immunotoxin action via direct inhibition of the toxin's enzymatic domain and therefore would not be appropriate for use in combination with PE-based immunotoxins. However it is possible that PARP inhibitors could serve as effective prophylactic treatments or even antidotes following exposures to pathogenic ADP-ribosylating toxins such as cholera toxin, diphtheria toxin, PE or others [26, 27].

## Discussion

Immunotoxins were championed as anti-cancer agents because of the reputed potency associated with the enzymatic domain of the bacterial toxin joined to the targeting antibody [2, 3]. However, clinical evaluation of several immunotoxins has demonstrated variable results ranging from a high rate of complete remissions in Hairy Cell Leukemia to more modest response rates for other B-cell malignancies including NHL, CLL and ALL [4–6, 28]. The reason for these disparate responses is currently poorly understood. While a detailed study of patient tumor cell biology might uncover why HCL cells are very sensitive to immunotoxin treatments while other B-cell malignancies are less so, a more pragmatic approach would seek out drugs that can convert resistant cells to sensitive ones. When mesothelin was targeted with the immunotoxin, SS1P, there were no objective responses from two early trials, one involving the continuous administration of agent and the other involving bolus infusions [7, 8]. Only when SS1P was administered in the presence of pemetrexed and cisplatin, was there evidence of objective responses [29]. Thus, there is clinical precedence for the use of drug combinations to enhance immunotoxin outcomes. While this is not a great surprise, as most cancer treatments use combination therapies to achieve superior outcomes, it was far from obvious which drugs would be best suited to accompany immunotoxin treatments.

In earlier published work we reported on individual compounds that exhibited immunotoxin enhancing activity [30–34]. Here we report on a much wider collection of compounds, those found within the MIPE-3 library. The library contains approximately 500 approved or near-approved small molecular weight drugs including many that are cancer-focused. Our screens allowed us to identify, both general enhancers of immunotoxin action and a number of cell-specific enhancers. We confirmed the action of three of these enhancers (salinomycin, nilotinib and everolimus) in more detail and then focused primarily on everolimus to establish mechanistic insights. Further all confirmation studies were carried out at concentrations of drug that are achievable in humans, thereby providing a rational basis for continued preclinical development. In this regard, we suggest that this approach has been quite successful in identifying useful candidates that might otherwise not have been uncovered via traditional investigator-driven experimentation. We note also that the level of enhancement with these agents was substantial, generally around 10-fold, suggesting that major inefficiencies exist in immunotoxin-mediated killing. While enhancing compounds were sought, we were aware that mitigators might also exist within the MIPE-3 collection. In fact, PARP inhibitors were identified as 'strong' mitigators and were found to block immunotoxin action as well as two native toxins, exhibiting ADP-ribosyl transferase activity. While humans are now rarely exposed to diphtheria toxin, periodic epidemics of cholera still arise, where the pathogenic action of cholera toxin is recognized as a major ADP-ribosylating virulence factor [27]. Because they are relatively non-toxic for humans, we speculate that PARP inhibitors could be useful agents in the treatment of diseases caused by bacterial ADP-ribosylating toxins.

Among the most notable enhancers was the mTORC1 inhibitor, everolimus, which demonstrated strong potentiation for several immunotoxins across multiple cell lines and exhibited strong inhibition of protein synthesis when combined with immunotoxins (Fig 3B). Further, data support a true synergism between everolimus and immunotoxins. This contention is supported by results showing the enhancement of immunotoxin activity at concentrations of everolimus that were non-cytotoxic, by themselves. Because of this synergism, we set out to explore the mechanistic underpinnings of this combination. Analysis of the protein levels and phosphorylation states of both S6K1 and 4EBP1 proved insightful. There was a synergistic blockade of S6K1 phosphorylation and down-regulation of S6K1 protein levels. The inhibition of 4EBP1 phosphorylation at multiple sites (including T37/46) was strongly enhanced in cells treated with both entities. The resulting block of eIF4E together with the inhibition of S6K1 activity offers a strong rationale for the observed inhibition of protein synthesis and the synergistic ability of this combination to kill cells. Analysis of tumor volume and percent survival of a KB3-1 xenograft model validated the utility of this drug combination and highlighted its potential for clinical utility (Fig 4). Interestingly, when we examined, in detail, the dose response curves of other inhibitors targeting the mTOR kinase specifically (and not proteins in the mTORC1 complex) we noted very little enhancing activity (Table 2). To extend the results obtained in the screens, we explored combination treatments with HB21-PE40 and KB3-1 cells in the presence of the following mTOR kinase inhibitors: Torin1, AZD-8055 and KU-0063794. Similar to the screening results, we saw no enhancement with these compounds, only weak mitigation (data not shown). We conclude that so-called 'gain of function' rapalogs (such as everolimus) but not mTOR kinase inhibitors, enhance immunotoxin action. Mechanistic insights that explain these disparate results are currently being sought.

Thematically similar to this small molecule screen, we recently conducted a genome-wide RNAi screen designed to identify cellular targets that either enhanced or mitigated immunotoxins action [35]. By comparing and contrasting the outcomes from these genetic and chemical screens, we can make several conclusions: first, both were valuable in their own right because they pointed to different kinds of targets—the RNAi screen highlighted the role of ARF proteins and elements of the ER/Golgi system that are not easily druggable, while the drug screen identified compounds already approved for human use that could be advanced to the clinic. Of interest, the activities of everolimus or nilotinib were not readily matched to down-regulated gene products. Regarding everolimus for instance, a review of our RNAi data found no strong sensitizing candidates in the mTOR/PI3K pathway. This could be due to a variety of reasons: some genes are difficult to target, some proteins are long lived and not amenable to RNAi and finally some pathways are complex where redundancy requires the silencing of more than one member to shut down a pathway. Regarding nilotinib, we noted that this drug, but not other BCR-Abl inhibitors enhanced, immunotoxin action. And consistent with that result, when we reviewed our RNAi data there was no evidence that Abl knockdowns enhanced immunotoxin action. However, nilotinib does target other kinases including DDR1 and the PDGF receptor [36]. We have published recently that DDR1 knockdowns can enhance immunotoxin action [37]. However, imatinib also targets DDR1 but does not enhance immunotoxins, so consistent connections between drug action and genetic targeting have yet to be established at least with regard to immunotoxin activity [37].

In summary, we set out on a mostly a translational enterprise where the primary goal was to identify compounds that could be advanced into the clinic in combination with immunotoxin treatments. We suggest that our screening of drugs and antibody-based therapeutics was a success because several enhancers were identified, including two approved compounds. Also, PARP inhibitors were found to be strong mitigators and should not be combined with immunotoxins. Technical problems did not allow for full matrix screening where both agents are varied over large concentrations. However, a curve shift approach was quite valuable and has provided us with value leads for preclinical development.

## Materials and Methods

### Reagents

Three immunotoxins, HB21PE40, HA22 and SS1P were produced recombinantly in E. coli as described previously [38]. Nilotinib and everolimus were purchased from Selleck Chemicals LLC (Houston, TX), dissolved in dimethyl sulphoxide (DMSO) at 10 mmol/L stock concentration, and stored frozen at −80°C. Salinomycin was from Sigma-Aldrich (St. Louis, MO) and was handled similarly.

### Cell Lines

The B cell precursor leukemia cell line Nalm6 was obtained from Alan Wayne (National Cancer Institute, Bethesda, MD). The cervical adenocarcinoma cell line KB3-1 was obtained from Michael Gottesman (National Cancer Institute, Bethesda, MD). The Burkitt's lymphoma cell lines, CA46 (CRL-1648) and Daudi (CCL-213), the Mantle Cell Lymphoma JeKo-1 (CRL-3006) and the triple negative breast cancer cell line MDA-MB-468 (HTB-132) were purchased from ATCC. Epithelial cells were grown in Dulbecco's Modified Eagle Medium (DMEM, Life Technology Grand Island, NY) plus 10% fetal bovine serum while hematological cell lines were grown in RPMI1640 (Life Technology Grand Island, NY) plus 10% fetal bovine serum.

### Cytotoxicity Assays

Cell viability was determined with the CellTiter-Glo Luminescent Cell Viability Assay kit (Promega, Madison WI). This assay quantifies the amount of ATP present, which indicates the presence of metabolically active cells. The primary screening assays with the MIPE-3 library were performed as described previously [9] and in a recently published technical report on ranking relative activities [39]. In the confirmation assays, ATP was measured as luminescence produced by the mono-oxygenation of luciferin catalyzed by the Ultra-Glo-luciferase. Protein synthesis inhibition was quantified by incubating cells in a 96-well format with 2 μCi/ml ^3^H-leucine (Perkin Elmer, Boston, MA) and counting samples on a filter mat using a Wallac Beta plate reader (Perkin Elmer, Boston MA).

### Immunoblot analysis

Treated or control cells were collected, washed with phosphate buffered saline solution (PBS), and solubilized in RIPA buffer (Thermo Scientific Pierce, Rockford, IL) with protease and phosphatase inhibitors (Roche Applied Science, Indianapolis, IN). Protein concentrations were determined using the Nanodrop2000c Spectrophotometer (Thermo Scientific, Waltham, MA). Equal amounts of protein were loaded onto NuPAGE 4–12% Bis-Tris gels (Life Technology Invitrogen, Grand Island, NY) and transferred to nitrocellulose membranes (Life Technology Invitrogen, Grand Island, NY). The following primary antibodies were used: p70S6K, pp70S6K (T389), 4EBP1, p4EBP1 (T37/46), p4EBP1 (T70), p4EBP1 (S65), mTOR, Raptor and Mcl-1 from Cell Signaling Technology, Danvers, MA; FKBP12 from Santa Cruz Biotechnology, Santa Cruz, CA; actin from BD Biosciences, San Jose, CA. Primary antibodies were routinely detected with donkey anti-mouse horseradish peroxidase or donkey anti-rabbit horseradish peroxidase (Jackson ImmunoResearch, West Grove, PA) using the SuperSignal West Pico Chemiluminescent Substrate kit (Thermo Scientific Pierce, Rockford, IL).

### KB3-1 xenograft model

All animal experiments were performed in accordance with NIH guidelines and approved by the NCI Animal Care and Use Committee. KB3-1 tumors were grown in female nude athymic mice (Charles River Laboratories). 5x10^6^ cells/mouse in serum free DMEM were mixed with Matrigel (Corning)(4 mg/mL) and injected into the rear flank of mice weighing 20-25g. After tumor volume had reached approximately 100mm^3^, mice were treated with vehicle alone, everolimus (5mg/kg), HB21-PE40 (3μg/mouse), or a combination of everolimus and HB21-PE40. Four injections were performed every other day, with HB21-PE40 (PBS + 0.2% human serum albumin) administered via tail vein and everolimus (Cremaphor EL and 20% 2-hydroxypropyl-beta-cyclodextrin) administered via IP injection. Tumor volumes and animal weights were measured at least three times weekly. Tumor volume was calculated as 0.5x(LxW^2^). Animals were sacrificed once tumors reached 1500mm^3^ or became necrotic. Time to endpoint was displayed on a Kaplan-Meyer plot. All statistics were performed in GraphPad Prism v5 software (GraphPad Software, Inc.). The tumor volumes were compared by two-way T-test at each condition endpoint (ie: Vehicle vs. all other conditions; Everolimus vs. HB21-PE40/Combo; HB21-PE40 vs. Combo) with p<0.05 significant. Key: *p<0.05, **p<0.01, ***p<0.001. The survival curves were compared by Log-Rank test, with p<0.05 significant. Key: *p<0.05, **p<0.01, ***p<0.001.

## Supporting Information

S1 Fig Confirmation study. A. Primary screening outcome for U-73122 and Abiraterone in KB3-1 and Nalm-6 cells. B. Primary screening outcome for Aurora kinase inhibitors tozasertib and SNS-314 in KB3-1 and Nalm-6 cells.
(EPS)

S2 Fig Confirmation study. A. Primary screening outcome for the PI3K inhibitor Wortmannin and the AKT inhibitor GSK-690693 in KB3-1 and the Nalm-6 cells. B. Primary screening results for Rucaparib and Veliparib in KB3-1 cells.
(EPS)

S3 Fig Viability of different cell lines treated with drug alone for the confirmation studies in Fig 2 and in Fig 3A. Two tailed unpaired t-tests were performed comparing the viability of no treated cells versus treated with the drug with p<0.05 significant. *p<0.05, **p<0.01, ***p<0.001.
(EPS)

S4 Fig Additional data on combination studies with everolimus. A. The primary screening outcome for Everolimus in the KB3-1 line. B. The pilot in vivo study utilizing everolimus at 10 mg/kg and the HB21PE40 immunotoxin at 0.15mg/kg. Tumor volumes were compared by unpaired two-tailed t-test at each measurement.
(EPS)

S1 Method Area Between the Curves (ABC) scoring method. (DOCX)

S1 Table Complete screening data. (XLSX)

## References

[1] Scott AM, Wolchok JD, Old LJ. Antibody therapy of cancer. Nat Rev Cancer. 2012;12(4):278–87. 10.1038/nrc3236 22437872

[2] Pastan I, Hassan R, Fitzgerald DJ, Kreitman RJ. Immunotoxin therapy of cancer. Nat Rev Cancer. 2006;6(7):559–65. 16794638 10.1038/nrc1891

[3] Pastan I, Hassan R, FitzGerald DJ, Kreitman RJ. Immunotoxin treatment of cancer. Annu Rev Med. 2007;58:221–37. 17059365 10.1146/annurev.med.58.070605.115320

[4] Kreitman RJ, Squires DR, Stetler-Stevenson M, Noel P, FitzGerald DJ, Wilson WH, et al Phase I trial of recombinant immunotoxin RFB4(dsFv)-PE38 (BL22) in patients with B-cell malignancies. Journal of clinical oncology: official journal of the American Society of Clinical Oncology. 2005;23(27):6719–29. 16061911 10.1200/JCO.2005.11.437

[5] Kreitman RJ, Stetler-Stevenson M, Margulies I, Noel P, Fitzgerald DJ, Wilson WH, et al Phase II trial of recombinant immunotoxin RFB4(dsFv)-PE38 (BL22) in patients with hairy cell leukemia. Journal of clinical oncology: official journal of the American Society of Clinical Oncology. 2009;27(18):2983–90. 19414673 10.1200/JCO.2008.20.2630 PMC2702232

[6] Kreitman RJ, Tallman MS, Robak T, Coutre S, Wilson WH, Stetler-Stevenson M, et al Phase I trial of anti-CD22 recombinant immunotoxin moxetumomab pasudotox (CAT-8015 or HA22) in patients with hairy cell leukemia. Journal of clinical oncology: official journal of the American Society of Clinical Oncology. 2012;30(15):1822–8. 22355053 10.1200/JCO.2011.38.1756 PMC3383181

[7] Hassan R, Bullock S, Premkumar A, Kreitman RJ, Kindler H, Willingham MC, et al Phase I study of SS1P, a recombinant anti-mesothelin immunotoxin given as a bolus I.V. infusion to patients with mesothelin-expressing mesothelioma, ovarian, and pancreatic cancers. Clinical cancer research: an official journal of the American Association for Cancer Research. 2007;13(17):5144–9. 17785569 10.1158/1078-0432.CCR-07-0869

[8] Kreitman RJ, Hassan R, Fitzgerald DJ, Pastan I. Phase I trial of continuous infusion anti-mesothelin recombinant immunotoxin SS1P. Clinical cancer research: an official journal of the American Association for Cancer Research. 2009;15(16):5274–9. 19671873 10.1158/1078-0432.CCR-09-0062 PMC2754261

[9] Mathews Griner LA, Guha R, Shinn P, Young RM, Keller JM, Liu D, et al High-throughput combinatorial screening identifies drugs that cooperate with ibrutinib to kill activated B-cell-like diffuse large B-cell lymphoma cells. Proceedings of the National Academy of Sciences of the United States of America. 2014;111(6):2349–54. 10.1073/pnas.1311846111 24469833 PMC3926026

[10] Saglio G, Kim DW, Issaragrisil S, le Coutre P, Etienne G, Lobo C, et al Nilotinib versus imatinib for newly diagnosed chronic myeloid leukemia. N Engl J Med. 2010;362(24):2251–9. 10.1056/NEJMoa0912614 20525993

[11] Rix U, Hantschel O, Durnberger G, Remsing Rix LL, Planyavsky M, Fernbach NV, et al Chemical proteomic profiles of the BCR-ABL inhibitors imatinib, nilotinib, and dasatinib reveal novel kinase and nonkinase targets. Blood. 2007;110(12):4055–63. 17720881 10.1182/blood-2007-07-102061

[12] Reichardt P, Montemurro M. Clinical experience to date with nilotinib in gastrointestinal stromal tumors. Semin Oncol. 2011;38 Suppl 1:S20–7. 10.1053/j.seminoncol.2011.01.015 21419932

[13] Gupta PB, Onder TT, Jiang G, Tao K, Kuperwasser C, Weinberg RA, et al Identification of selective inhibitors of cancer stem cells by high-throughput screening. Cell. 2009;138(4):645–59. 10.1016/j.cell.2009.06.034 19682730 PMC4892125

[14] King TD, Suto MJ, Li Y. The Wnt/beta-catenin signaling pathway: a potential therapeutic target in the treatment of triple negative breast cancer. Journal of cellular biochemistry. 2012;113(1):13–8. 10.1002/jcb.23350 21898546 PMC10924801

[15] Callaway TR, Edrington TS, Rychlik JL, Genovese KJ, Poole TL, Jung YS, et al Ionophores: their use as ruminant growth promotants and impact on food safety. Current issues in intestinal microbiology. 2003;4(2):43–51. 14503688

[16] Ortolani S, Ciccarese C, Cingarlini S, Tortora G, Massari F. Suppression of mTOR pathway in solid tumors: lessons learned from clinical experience in renal cell carcinoma and neuroendocrine tumors and new perspectives. Future Oncol. 2015;11(12):1809–28. 10.2217/fon.15.81 26075448

[17] Steelman LS, Martelli AM, Cocco L, Libra M, Nicoletti F, Abrams SL, et al The Therapeutic Potential of mTOR Inhibitors in Breast Cancer. Br J Clin Pharmacol. 2016. 10.1111/bcp.12958 PMC5061784 27059645

[18] Atkins MB, Yasothan U, Kirkpatrick P. Everolimus. Nature reviews Drug discovery. 2009;8(7):535–6. 10.1038/nrd2924 19568281

[19] Magnuson B, Ekim B, Fingar DC. Regulation and function of ribosomal protein S6 kinase (S6K) within mTOR signalling networks. The Biochemical journal. 2012;441(1):1–21. 10.1042/BJ20110892 22168436

[20] Hara K, Yonezawa K, Kozlowski MT, Sugimoto T, Andrabi K, Weng QP, et al Regulation of eIF-4E BP1 phosphorylation by mTOR. The Journal of biological chemistry. 1997;272(42):26457–63. 9334222 10.1074/jbc.272.42.26457

[21] Andersson Y, Juell S, Fodstad O. Downregulation of the antiapoptotic MCL-1 protein and apoptosis in MA-11 breast cancer cells induced by an anti-epidermal growth factor receptor-Pseudomonas exotoxin a immunotoxin. International journal of cancer Journal international du cancer. 2004;112(3):475–83. 15382075 10.1002/ijc.20371

[22] Antignani A, Sarnovsky R, FitzGerald DJ. ABT-737 promotes the dislocation of ER luminal proteins to the cytosol, including pseudomonas exotoxin. Molecular cancer therapeutics. 2014;13(6):1655–63. 10.1158/1535-7163.MCT-13-0998 24739394 PMC6258188

[23] Kang SA, Pacold ME, Cervantes CL, Lim D, Lou HJ, Ottina K, et al mTORC1 phosphorylation sites encode their sensitivity to starvation and rapamycin. Science. 2013;341(6144):1236566 10.1126/science.1236566 23888043 PMC3771538

[24] Turgeon Z, Jorgensen R, Visschedyk D, Edwards PR, Legree S, McGregor C, et al Newly discovered and characterized antivirulence compounds inhibit bacterial mono-ADP-ribosyltransferase toxins. Antimicrob Agents Chemother. 2011;55(3):983–91. 10.1128/AAC.01164-10 21135177 PMC3067067

[25] Wei H, Xiang L, Wayne AS, Chertov O, FitzGerald DJ, Bera TK, et al Immunotoxin resistance via reversible methylation of the DPH4 promoter is a unique survival strategy. Proceedings of the National Academy of Sciences of the United States of America. 2012;109(18):6898–903. 10.1073/pnas.1204523109 22509046 PMC3345006

[26] Simon NC, Aktories K, Barbieri JT. Novel bacterial ADP-ribosylating toxins: structure and function. Nature reviews Microbiology. 2014;12(9):599–611. 10.1038/nrmicro3310 25023120 PMC5846498

[27] Deng Q, Barbieri JT. Molecular mechanisms of the cytotoxicity of ADP-ribosylating toxins. Annual review of microbiology. 2008;62:271–88. 10.1146/annurev.micro.62.081307.162848 18785839

[28] Wayne AS, Kreitman RJ, Findley HW, Lew G, Delbrook C, Steinberg SM, et al Anti-CD22 immunotoxin RFB4(dsFv)-PE38 (BL22) for CD22-positive hematologic malignancies of childhood: preclinical studies and phase I clinical trial. Clinical cancer research: an official journal of the American Association for Cancer Research. 2010;16(6):1894–903. 20215554 10.1158/1078-0432.CCR-09-2980 PMC2840067

[29] Hassan R, Sharon E, Thomas A, Zhang J, Ling A, Miettinen M, et al Phase 1 study of the antimesothelin immunotoxin SS1P in combination with pemetrexed and cisplatin for front-line therapy of pleural mesothelioma and correlation of tumor response with serum mesothelin, megakaryocyte potentiating factor, and cancer antigen 125. Cancer. 2014;120:3311–9. 10.1002/cncr.28875 24989332 PMC6334650

[30] Traini R, Ben-Josef G, Pastrana DV, Moskatel E, Sharma AK, Antignani A, et al ABT-737 overcomes resistance to immunotoxin-mediated apoptosis and enhances the delivery of pseudomonas exotoxin-based proteins to the cell cytosol. Molecular cancer therapeutics. 2010;9(7):2007–15. 10.1158/1535-7163.MCT-10-0257 20587662 PMC2943340

[31] Du X, Xiang L, Mackall C, Pastan I. Killing of resistant cancer cells with low Bak by a combination of an antimesothelin immunotoxin and a TRAIL Receptor 2 agonist antibody. Clinical cancer research: an official journal of the American Association for Cancer Research. 2011;17(18):5926–34. 10.1158/1078-0432.CCR-11-1235 PMC3177016 21813632

[32] Mattoo AR, FitzGerald DJ. Combination treatments with ABT-263 and an immunotoxin produce synergistic killing of ABT-263-resistant small cell lung cancer cell lines. International journal of cancer Journal international du cancer. 2013;132(4):978–87. 10.1002/ijc.27732 22821746 PMC3527691

[33] Mattoo AR, Pastan I, Fitzgerald D. Combination treatments with the PKC inhibitor, enzastaurin, enhance the cytotoxicity of the anti-mesothelin immunotoxin, SS1P. PloS one. 2013;8(10):e75576 10.1371/journal.pone.0075576 24130723 PMC3794001

[34] Hollevoet K, Antignani A, Fitzgerald DJ, Pastan I. Combining the antimesothelin immunotoxin SS1P with the BH3-mimetic ABT-737 induces cell death in SS1P-resistant pancreatic cancer cells. Journal of immunotherapy. 2014;37(1):8–15. 10.1097/CJI.0000000000000010 24316551 PMC7667488

[35] Pasetto M, Antignani A, Ormanoglu P, Buehler E, Guha R, Pastan I, et al Whole-genome RNAi screen highlights components of the endoplasmic reticulum/Golgi as a source of resistance to immunotoxin-mediated cytotoxicity. Proceedings of the National Academy of Sciences of the United States of America. 2015;112(10):E1135–42. 10.1073/pnas.1501958112 25713356 PMC4364196

[36] Davis MI, Hunt JP, Herrgard S, Ciceri P, Wodicka LM, Pallares G, et al Comprehensive analysis of kinase inhibitor selectivity. Nature biotechnology. 2011;29(11):1046–51. 10.1038/nbt.1990 22037378

[37] Ali-Rahmani F, FitzGerald DJ, Martin S, Patel P, Prunotto M, Ormanoglu P, et al Anticancer Effects of Mesothelin-Targeted Immunotoxin Therapy Are Regulated by Tyrosine Kinase DDR1. Cancer research. 2015;76:1560–8. 10.1158/0008-5472.CAN-15-2401 26719540 PMC4794364

[38] Onda M. Recombinant immunotoxins with low endotoxins for clinical and animal studies. Methods Mol Biol. 2012;907:627–43. 10.1007/978-1-61779-974-7_35 22907377

[39] Guha R, Mathews Griner LA, Keller JM, Zhang X, Fitzgerald D, Antignani A, et al Ranking Differential Drug Activities from Dose-Response Synthetic Lethality Screens. Journal of biomolecular screening. 2016;in press. 10.1177/1087057116644890 PMC7876566 27112173

