# Supplementary material for: Chemical Screens Identify Drugs that Enhance or Mitigate Cellular Responses to Antibody-Toxin Fusion Proteins
Source: PLoS One. 2016 Aug 24;11(8):e0161415. doi: 10.1371/journal.pone.0161415 (PMC4996465; doi:10.1371/journal.pone.0161415)
Supplement: S1 Method — (DOCX) [file pone.0161415.s005.docx]

**Supporting Information for…**

**Chemical Screens Identify Drugs that Enhance or Mitigate Cellular Responses to Immunotoxin Treatment**

Antonella Antignani^1a^, Lesley Mathews Griner^2a^, Rajarshi Guha^2^,Nathan Simon^1^, Matteo Pasetto^1^, Jonathan Keller^2^, Manjie Huang^1^, Evan Angelus^1^, Ira Pastan^1^, Marc Ferrer^2^, David J. FitzGerald^1^* and Craig J. Thomas^2^*.

^1^Laboratory of Molecular Biology, Center for Cancer Research, National Cancer Institute, National Institutes of Health, Bethesda, Maryland 20892-4264

^2^Division of Preclinical Innovation, National Center for Advancing Translational Sciences, National Institutes of Health, Rockville, Maryland, 20850.

^a^Antonella Antignani and Lesley A. Matthews-Griner contributed equally to this work.

Corresponding authors*:

David FitzGerald, Laboratory of Molecular Biology, National Cancer Institute, 37 Convent Drive, Room 5124, Bethesda, MD 20892-4264, USA.

Tel: (301) 496-9457; Fax: (301) 402-1344; e-mail: [fitzgerd@helix.nih.gov](mailto:pastani@mail.nih.gov)

Craig J. Thomas, Division of Preclinical Innovation, National Center for Advancing Translational Sciences, National Institutes of Health, Rockville, Maryland, 20850, USA.

Tel (301) 217-4079; email [craigt@mail.nih.gov](mailto:craigt@mail.nih.gov)

**S1 Method. Area Between the Curves (ABC) scoring method**. The area under the curve (AUC) for each dose response was computed using the trapezoidal rule [REF: Atkinson, Kendall E. (1989), An Introduction to Numerical Analysis (2nd ed.), New York: John Wiley & Sons). The ABC was then calculated as the difference between the AUCs for the untreated and immunotoxin treated samples. Thus, negative values indicate a shift of the dose response curve for the treated sample to the left and thus a sensitization of the sample by the immunotoxin. Importantly, the ABC score can be confounded by toxicity effects. In general, toxic compounds will tend to exhibit low AUC’s and thus can be confused with highly potent compounds (this is especially true when the compound is highly cytotoxic and exhibits little to no dose response).
